# Characterization of Three Complete Mitogenomes of Flatidae (Hemiptera: Fulgoroidea) and Compositional Heterogeneity Analysis in the Planthoppers’ Mitochondrial Phylogenomics

**DOI:** 10.3390/ijms22115586

**Published:** 2021-05-25

**Authors:** Deqiang Ai, Lingfei Peng, Daozheng Qin, Yalin Zhang

**Affiliations:** 1 Key Laboratory of Plant Protection Resources & Pest Management of the Ministry of Education, College of Plant Protection, Northwest A&F University, Yangling 712100, Shaanxi, China; aideqiang@nwafu.edu.cn; 2 Key Laboratory of Integrated Pest Management for Fujian-Taiwan Crops, Ministry of Agriculture, Fujian Ag-riculture and Forestry University, Fuzhou 350002, Fujian, China; lingfeipeng@fafu.edu.cn

**Keywords:** flatidae, planthopper phylogeny, mitochondrial phylogenomics, sequence heterogeneity, site-heterogeneous mixture model

## Abstract

Although sequences of mitogenomes have been widely used for investigating phylogenetic relationship, population genetics, and biogeography in many members of Fulgoroidea, only one complete mitogenome of a member of Flatidae has been sequenced. Here, the complete mitogenomes of *Cerynia lineola*, *Cromna sinensis,* and *Zecheuna tonkinensis* are sequenced. The gene arrangements of the three new mitogenomes are consistent with ancestral insect mitogenomes. The strategy of using mitogenomes in phylogenetics remains in dispute due to the heterogeneity in base composition and the possible variation in evolutionary rates. In this study, we found compositional heterogeneity and variable evolutionary rates among planthopper mitogenomes. Phylogenetic analysis based on site-homogeneous models showed that the families (Delphacidae and Derbidae) with high values of Ka/Ks and A + T content tended to fall together at a basal position on the trees. Using a site-heterogeneous mixture CAT + GTR model implemented in PhyloBayes yielded almost the same topology. Our results recovered the monophyly of Fulgoroidea. In this study, we apply the heterogeneous mixture model to the planthoppers’ phylogenetic analysis for the first time. Our study is based on a large sample and provides a methodological reference for future phylogenetic studies of Fulgoroidea.

## 1. Introduction

The mitochondrion is a tiny eukaryotic organelle that generates almost all of an organism’s energy in the form of ATP [1,2]. Its functions are closely involved in energy, sex, fertility, apoptosis, aging, and death in the organism [2]. The mitochondrial genomes of insects usually comprise 37 encoding genes: 13 protein-encoding genes (PCGs), 22 transfer RNA genes (tRNAs), two ribosomal RNA genes (rRNAs), and a control region (CR) or A + T-rich region on a circular double-stranded molecule [1,3]. Sequences of mitogenomes are now widely used for the study of phylogenetic relationships, population genetics, and biogeography [3,4,5,6].

The superfamily Fulgoroidea comprises 34 families with more than 9000 species worldwide [7]. All species in Fulgoroidea are phytophagous, and some of them are recognized as ecologically and economically significant pests of main agricultural crops, such as corn, wheat, rice, and barley. This makes the phylogeny of the Fulgoroidea of considerable interest to biologists. However, the phylogenetic relationships among these planthopper families still remain uncertain based on earlier morphological and molecular analyses [8,9,10]. Recently, mitogenome data have been more widely applied to studies of planthopper phylogeny based on the lower costs of high-throughput sequencing [11,12,13,14,15].

Flatidae are one of the largest families within the superfamily Fulgoroidea (Hemiptera: Auchenorrhyncha) with 1446 species and 289 genera within the two subfamilies: Flatinae Spinola, 1839 and Flatoidinae Melichar, 1901. They are cosmopolitan in their distribution, with their highest diversity in the tropics and warm temperate areas. Flatids are all terrestrial and sap-sucking phytophagous insects with efficient mouthparts highly modified for extracting the liquid contents of plants. Some flatids are recognized as serious pests of economically important plants, such as coffee, tea, orange, cacao, pears, and other crops [16,17]. However, the mitogenome sequence of Flatidae was still lacking. Only one complete mitogenome sequence (*Geisha distinctissima*) has been reported [12]. In our study, we sequenced the complete mitogenomes of *Cerynia lineola*, *Cromna sinensis*, and *Zecheuna tonkinensis* and analyzed their mitochondrial structure.

Mitogenomes of insects have a high A + T content; base composition and evolutionary rate vary between lineages [18,19,20]. Compositional heterogeneity and variable evolutionary rates are often associated with systematic errors in the phylogenetic analysis [1]. Thus, the use of mitogenomes in phylogenetics remains disputed. In order to reduce the compositional bias of mitogenome sequences, the third codon positions of protein-coding genes were usually removed in the early phylogenetic analysis based on mitogenomes; however, this may lead to a significant loss of signal and affect nodal support [1,21]. The site-heterogeneous CAT model is able to adapt to the complexity present in the data, and it calculates the posterior mean number of classes to estimate the substitutional heterogeneity [22]. This model has been used widely for phylogenetic analyses using mitogenome sequences and has a positive impact in reducing the influence of compositional and mutational bias in mitochondrial phylogenomics [18,19,20,23]. The CAT model has been more suitable than other analyses in reconstructing expected relationships of major clades comprising higher and basal groups in the orders within Holometabola [19], in the suborder of Heteroptera [18], and Hemiptera [20]. In this study, the compositional heterogeneity, evolutionary rate, and A + T content of planthoppers’ mitogenome sequences were analyzed, and a phylogeny of Fulgoroidea was reconstructed based on current mitogenome data. The aim of this research is to analyze the effects of compositional heterogeneity on phylogenetic reconstruction based on planthopper mitogenome data, to provide a methodological reference, and to improve our understanding of the evolution of Fulgoroidea.

## 2. Results and Discussion

### 2.1. Mitogenome Organization and Base Composition

**Figure 1:**
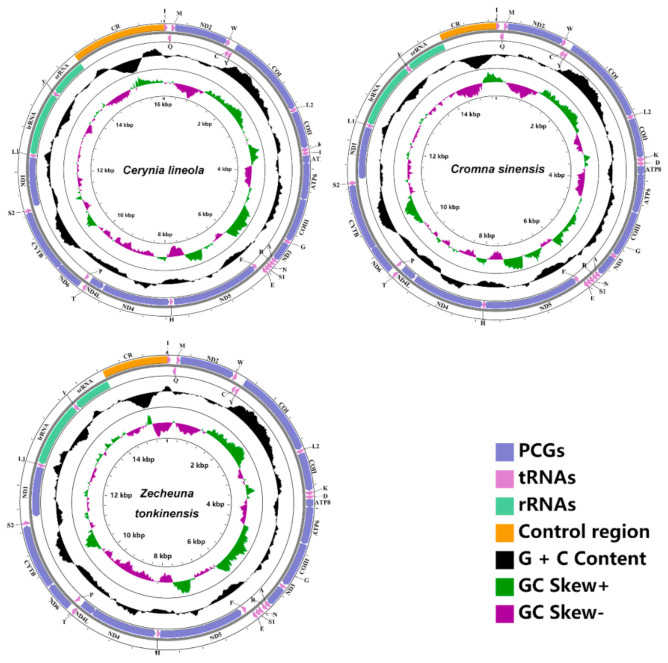
The mitogenomes of Cerynia lineola, Cromna sinensis and Zecheuna tonkinensis.

The mitogenomes of *Cerynia lineola*, *Cromna sinensis*, and *Zecheuna tonkinensis* were 16,053 bp, 15,329 bp, and 15,613 bp, respectively (Figure 1). Comparing these three new Flatidae species’ mitogenomes with the other reported Fulgoroidea mitogenomes, *Paracatonidia* sp. (Achilidae) had the smallest mitogenome with 15,214 bp [14] and *Nilaparvata lugens* (Delphacidae) contained the largest mitogenome at 17,619 bp [24]. This variation in the size of mitogenomes of planthoppers is mainly due to the variable size of the control region. The three newly sequenced mitogenomes of Flatidae included the 37 typical insect mitochondrial genes (13 PCGs, 22 tRNA genes, and 2 rRNA genes) and one non-coding region (CR). Among the 37 mitochondrial genes of these three new mitogenomes, the gene arrangement is consistent with the ancestral insect mitogenomes [1].

Nine PCGs and 14 tRNA genes were located on the majority strand (J-strand), whereas the other 14 genes (four PCGs, eight tRNA genes, and two rRNA genes) were located on the minority strand (N-strand) (Table S1–S3). The overall base composition of *Cerynia lineola* was A (47.6%), T (28.6%), G (8.4%), and C (15.4%); *Cromna sinensis* was A (47.4%), T (27.5%), G (9.3%), and C (15.8%); and A (49.0%), T (30.5%), G (7.5%), and C (13.0%) in *Zecheuna tonkinenesis* (Table S4). This exhibited the three mitogenomes with a high A+T content for the whole sequence reaching 76.2% in *Cerynia lineola*, 74.9% in *Cromna sinensis*, and 79.5% in *Zecheuna tonkinensis*. All three mitogenomes presented a positive AT skew and a negative GC skew. This situation is congruent with other insect mitogenomes [25].

### 2.2. Proteins-coding Genes and Codon Usage

**Figure 2:**
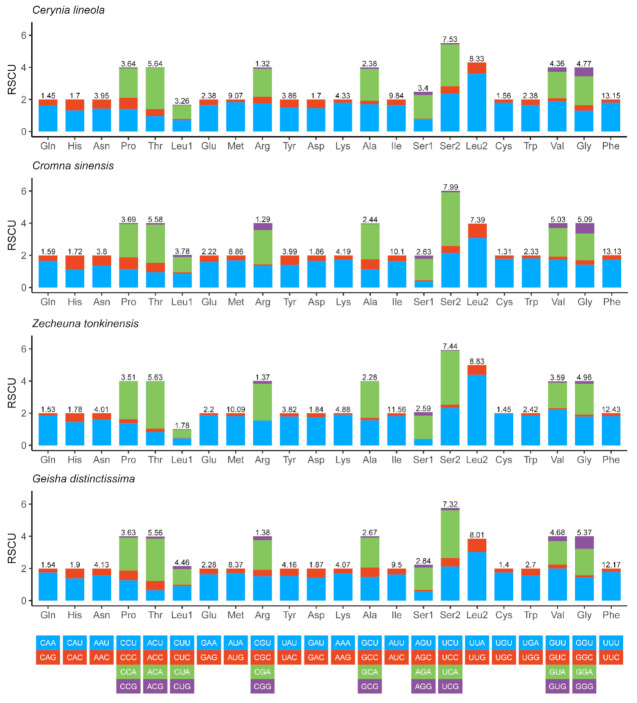
Relative synonymous codon usage (RSCU) of the mitogenomes of four Flatidae species.

The total length of the PCGs of the *Cerynia lineola*, *Cromna sinensis,* and *Zecheuna tonkinensis* was 10,986 bp, 10,998 bp, and 10,803 bp, respectively. All three newly sequenced Flatidae mitogenomes had a negative AT skew and negative GC skew in PCGs. The third codon position had the highest AT content. The second codon position had the lowest AT content. All PCGs in the three new mitogenomes started with the codon ATN (ATA/T/G). Most PCGs of these three mitogenomes stopped with the complete termination codon TAA or TAG, while the incomplete stop codon single T was used by *atp6* in three new mitogenomes and *nad5* in *Cromna sinensis*. In all four Flatidae mitogenomes, the termination TAA occurred more than TAG, and the single T was used the least in Flatidae. This incomplete termination codon has been observed in several other planthopper mitogenomes. The relative synonymous codon usage (RSCU) for four complete Flatidae mitogenomes is shown in Figure 2. The codon usage analysis shows that the third codon positions were more likely A or T than G or C. The most frequently used codons were UUA (Leu2), AUA (Met), AUU (Ile), and UUU (Phe), all composed of just A or U. The codon usage in these four Flatidae is consistent with usage in other planthoppers [13,14,24]. In addition, the codons CCG (Pro), ACG (Thr), and GCG (Ala) were missing in *Zecheuna tonkinensis*. Such missing codons have been detected in many other planthoppers [13,24].

### 2.3. Transfer and Ribosomal RNA Genes

The 22 transfer RNA genes of these three newly sequenced complete mitogenomes were dispersed discontinuously across the entire mitogenome. All 22 tRNA genes were identified in the same relative genomic positions as in the ancestral insect mitogenomes and *Geisha distinctissima* [1,12]. The size of tRNAs gene sequences ranged from 58 bp (*trnS1*) to 72 bp (*trnV*) in *Cerynia lineola* (Table S1), from 59 bp (*trnS1*) to 71 bp (*trnV*) in *Cromna sinensis* (Table S2), and from 58 bp (*trnS1*) to 69 bp (*trnQ*, *trnK*, and *trnF*) in *Zecheuna tonkinensis* (Table S3). The whole tRNA region of these three mitogenomes was 1406 bp in *Cerynia lineola*, 1399 bp in *Cromna sinensis*, and 1431 bp in *Zecheuna tonkinensis* (Table S4). The AT and GC skew values in tRNAs of these three mitogenomes were positive. The secondary structure of 21 tRNAs could be folded into the typical cloverleaf structure, except for the *trnS1* (AGN) with a reduced DHU arm that forms a simple loop (Figures S1–S3). This lack of the DHU arm in *trnS1* has been observed in most metazoans [26]. The mismatched types of unpaired base in these three new mitogenomes were G-U, A-A, U-U, A-C, A-G, U-C, G-G, and single A. A total of twenty-nine mismatched G-U, ten mismatched A-A, seven mismatched U-U, three mismatched A-C, two mismatched A-G, two single A, one mismatched U-C, and one mismatched G-G were found in *Cerynia lineola*. Twenty-five mismatched G-U, seven mismatched U-U, five mismatched A-A, three mismatched A-C, two single A, one mismatched U-C, and one mismatched U-U were found in *Cromna sinensis*. Further, seventeen mismatched G-U, nine mismatched U-U, eight mismatched A-A, three mismatched A-C, two single A, and one mismatched A-G were found in *Zecheuna tonkinensis*. The mismatched base pairs were also detected in other planthoppers families [13,14].

In all three new complete mitogenomes, the two ribosomal RNA genes were encoded on the N-strand. The size of *rrnL* was 1212 bp in *Cerynia lineola*, 1198 bp in *Cromna sinensis*, and 1229 bp in *Zecheuna tonkinensis*, all located between *trnL1* and *trnV*. The size of rrnS was 725 bp in *Cerynia lineola*, 740 bp in *Cromna sinensis*, and 732 bp in *Zecheuna tonkinensis*, all located between *trnV* and the control region. Both *rrnL* and *rrnS* had a negative AT skew and positive GC skew in these three mitogenomes.

### 2.4. Control Region

**Figure 3:**
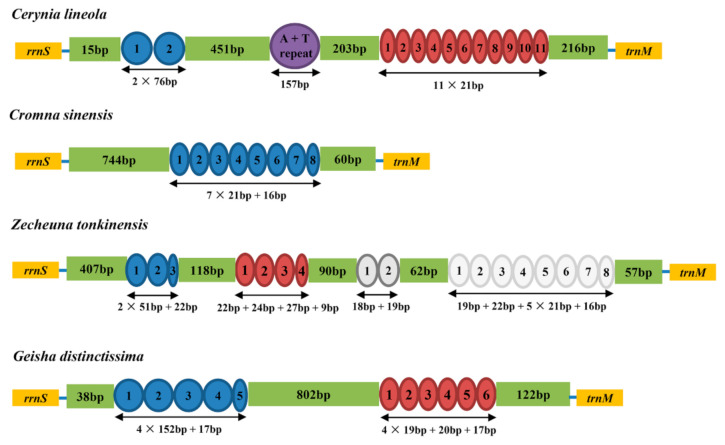
Organization of the control region in Flatidae mitogenomes. The blue, red, and gray ovals indicate the tandem repeats; the non-repeat regions are shown with green boxes.

The control region, alternatively called the A + T rich region or major non-coding region, is the longest non-coding sequence. All CR in these three new mitogenomes were located between *rrnS* and *trnI*. A total of 1706 bp in this control region were detected in the mitogenome of *Cerynia lineola*, 967 bp in *Cromna sinensis*, and 1139 bp in *Zecheuna tonkinensis*. Among these three Flatidae species, the nucleotide composition of the CR showed a positive AT skew and a negative GC skew: the AT content was 79.3% in *Cerynia lineola*, 80.6% in *Cromna sinensis*, and 83.6% in *Zecheuna tonkinensis*. Three types of tandem repeat regions were detected in the CR of *Cerynia lineola*, one type of tandem repeat region was detected in the CR of *Cromna sinensis*, four types of tandem repeat regions were detected in the CR of *Zecheuna tonkinensis,* and two types of tandem repeat regions were detected in the CR of *Geisha distinctissima* (Figure 3). This result is similar to other planthoppers [12,13,27]. The fragment length and copy number of tandem repeat regions among the CR of planthoppers mitogenomes indicates a considerable divergence.

### 2.5. Heterogeneous Sequence Divergence within Planthopper Mitogenomes

**Figure 4:**
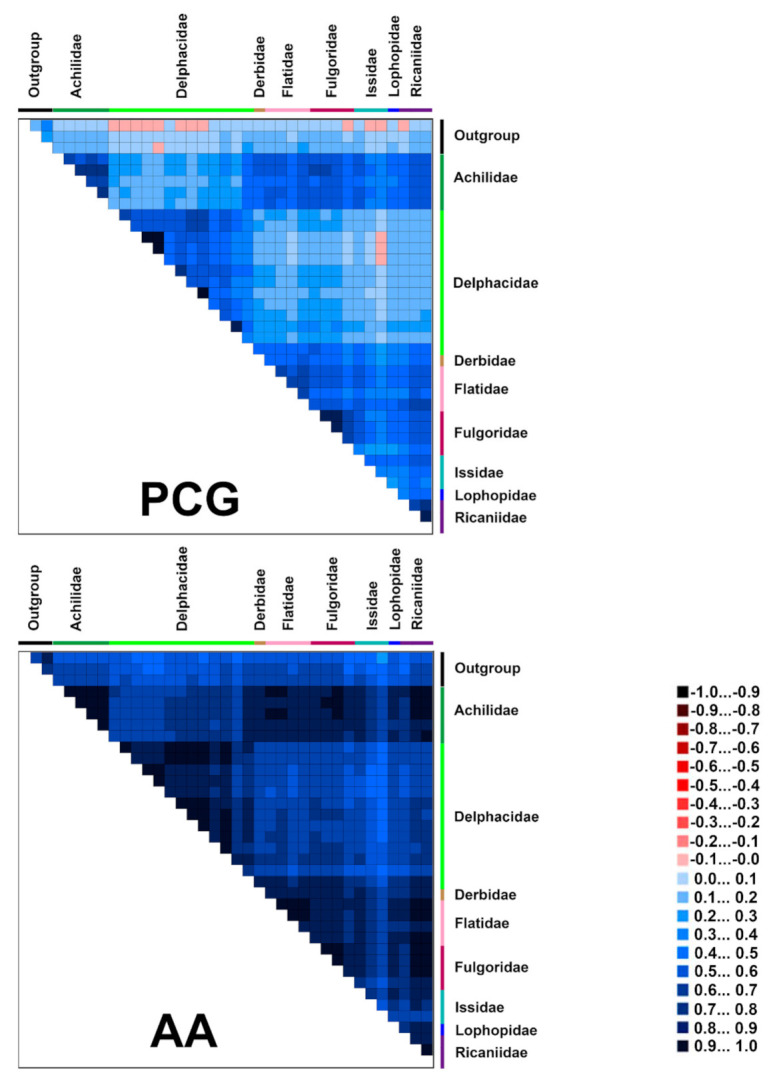
Heterogeneous sequence divergence within datasets PCG and AA of planthopper mitogenomes. The mean similarity score between sequences is represented by a colored square. AliGROOVE scores ranging from −1 (indicating great difference in rates from the remainder of the dataset; red coloring shows the significant heterogeneity) to +1 (indicating rates match in all other comparisons).

The analysis of the heterogeneity of the sequence divergence indicated that members of Delphacidae show remarkably higher heterogeneity than other planthoppers (the similar scores for pairwise sequence comparisons were the lowest) (Figure 4). In addition, the mitogenomes of Derbidae presented more heterogeneity than other planthopper groups. The degrees of heterogeneity of the PCG were higher than AA datasets (the other datasets were intermediate) (Figure S4). The third codon positions showed distinctly higher heterogeneity than the first and second codon positions. Hence, the datasets which excluded the third codon positions (PCG12 and PCG12RNA) also reduced the degree of sequence heterogeneity.

**Figure 5:**
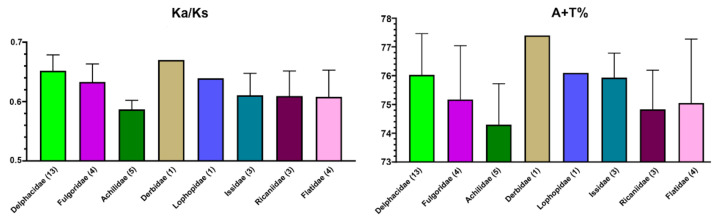
Ka/Ks ratio and A + T content for each family were calculated from the protein-coding genes only. Error bars represent standard deviations in data from multiple species.

We calculated Ka/Ks for each taxon with their outgroup (Figure 5) (Table S5). The value of Ka/Ks was obviously higher for Delphacidae (0.61–0.68) and Derbidae (0.67). These results showed that Delphacidae and Derbidae have a relatively higher evolutionary rate among Fulgoroidea. We also measured the compositional diversity of nucleotides of mitochondrial protein-coding genes across planthoppers families. A + T content had the nearly same tendency as the value of Ka/Ks. The sequences of Delphacidae and Derbidae were comparatively A + T richer than other families. Flatidae and Ricaniidae showed the lowest A + T content. Our analysis shows compositional heterogeneity among Fulgoroidea.

### 2.6. Phylogenetic Reconstruction Using Site-homogenous Models

**Figure 6:**
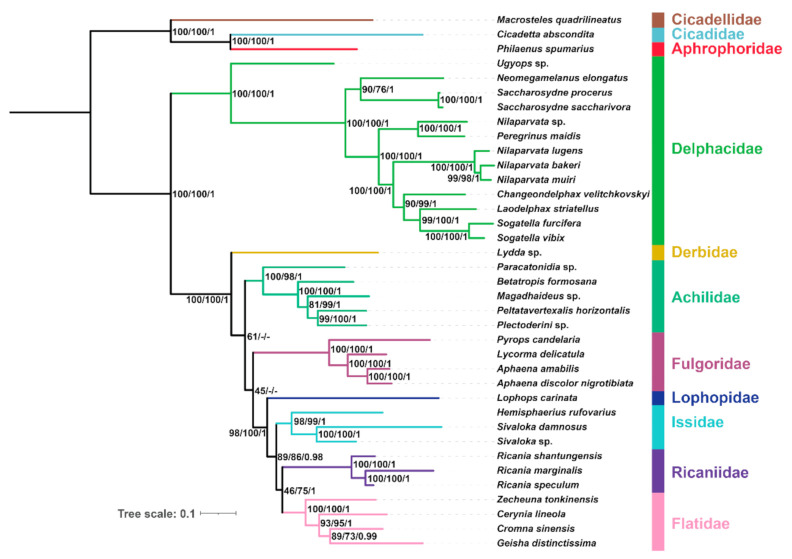
Phylogenetic tree inferred from IQ-TREE and MrBayes based on the datasets of AA and PCG12. Supports at nodes (from left to right) are ML bootstrap support values (BS) for AA, PCG12, and then Bayesian posterior probabilities (PP) for PCG12. “-” indicates the clades are different.

**Figure 7:**
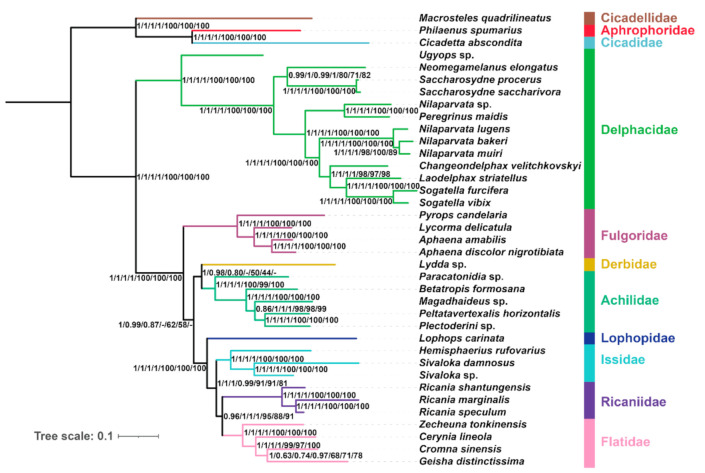
Phylogenetic tree inferred from IQ-TREE and MrBayes based on the datasets of AA, PCGRNA, PCG, and PCG12RNA. Supports at nodes (from left to right) are Bayesian posterior probabilities (PP) for AA, PCGRNA, PCG, PCG12RNA, and then ML bootstrap support values (BS) for PCGRNA, PCG, PCG12RNA. “-” indicates the clades are different.

The phylogenetic analyses of planthopper relationships all yielded very similar topologies, no matter which dataset, portioning scheme (Tables S6 and S7), or inference algorithm was used (Figure 6 and Figure 7). The monophyly of eight planthopper families was strongly supported in all analyses. Planthoppers were divided into two main groups, and Delphacidae was recovered as the sister group to the remaining families with strong support values (BS = 100; PP = 1). In all analyses under site-homogenous models, Fulgoridae, Achilidae, and Derbidae were more basal than Lophopidae, Issidae, Ricaniidae, and Flatidae, which is consistent with early morphological [28], nuclear molecular markers [10], and mitochondrial 16S rDNA Sequences [29] phylogenetic studies of Fulgoroidea. The topologies produced with the AA dataset based on ML analysis and the PCG12 dataset under ML and BI analysis were congruent: Derbidae was more basal than Achilidae and Fulgoridae, which has been recovered by early molecular phylogenetic analysis, used transcriptomes [8] and nuclear molecular markers [10] based on site homogeneity. In the remaining analyses, Fulgoridae was placed in a more basal position relative to Derbidae and Achilidae in the tree topologies. In a previous mitochondrial analysis, Fulgoridae had a relatively basal placement in the planthoppers [30].

Lophopidae, Issidae, Ricaniidae, and Flatidae formed a clade with strong branch support (BS > 98; PP = 1) by all analyses using site-homogenous models. Issidae had a close phylogenetic relationship with Flatidae. Ricaniidae had a middle to high support value (BS > 86; PP > 0.98). Flatidae was sister to Ricaniidae with moderate branch support (BS > 75; PP > 0.95) except ML analysis under AA dataset had low support (BS = 46). This indicates that all analyses based on site-homogenous analysis agree with previous studies [8,9,13,14].

### 2.7. Phylogenetic Reconstruction under Site-heterogeneous Mixture Model

**Figure 8:**
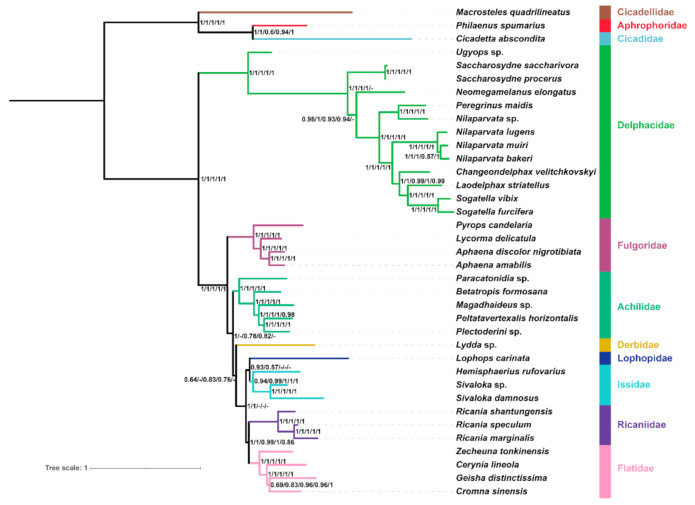
Phylogenetic tree inferred from PhyloBayes based on the datasets of PCG, PCGRNA, PCG12, PCG12RNA, and AA. Supports at nodes (from left to right) are Bayesian posterior probabilities (PP) for PCG, PCGRNA, PCG12, PCG12RNA, and AA. “-” indicates the clades are different.

The tree topologies under BI analysis of all datasets using the CAT + GTR model indicated that the eight planthoppers families (all selected families) were monophyletic (Figure 8). Delphacidae was the sister group to the remaining families with strong support values (PP = 1), and Fulgoridae was placed in a more basal portion than the other six families with high support (PP = 1). Derbidae tended to form a clade with Lophopidae, Issidae, Ricaniidae, and Flatidae except in the AA and PCGRNA datasets. The relationship between Issidae, Ricaniidae, and Flatidae was consistent with our analysis using site-homogenous models. In general, the use of the CAT + GTR model produced almost congruent topologies over site-homogeneous models in the phylogenetic analysis of planthoppers based on mitogenomes.

All datasets placed Fulgoridae in a relatively basal portion with a strong support value. This polygenetic relationship is a rare placement in comparison with previous morphological and molecular data [8,9,28,29]. Derbidae was placed in a more nested portion in this phylogeny, which is consistent with Song and Liang [9]. Planthoppers are a highly diverse insect group, and our taxon sampling is limited. Future research is needed with greater sampling to reconstruct the phylogenetic relationship among planthoppers and verify the influence of sequence compositional heterogeneity on the planthoppers’ mitochondrial phylogenomics.

### 2.8. Comparative Analysis of Site-homogenous and Site-heterogeneous Mixture Models

In all of our phylogenetic analysis, the relationship within Flatidae was (Selizini + (Ceryniini + (Phyllyphantini + Lawaini))). The closer relationship between Phyllyphantini and Lawaini had high support, which has also been observed in a previous morphological phylogenetic study [31].

Sequences compositional heterogeneity was detected within outgroup and ingroup (Delphacidae and Derbidae), especially in the datasets PCG and PCGRNA. Meanwhile, in the previous Hemiptera phylogenetic analysis, Fulgoroidea showed high compositional heterogeneity [20]. Thus, we infer that sequence compositional biases may be the cause of conflicting topologies between different analyses under site-homogenous models.

Within the ingroup, we mapped the value of Ka/Ks and A + T content onto the phylogenetic trees produced by the AA dataset based on ML analysis and the PCG12 dataset under ML and BI analyses. We observed that the families with high values of Ka/Ks and A + T content tended to fall together at the basal position on the trees. To reduce the impact of sequence compositional heterogeneity and mutational bias on phylogenetic analysis, we selected datasets that contained RNA data, excluded the third codon position of PCGs, and used amino acids data to improve the topologies under site-homogeneous models. The effect of these methods was limited. Sequence compositional heterogeneity still existed in these datasets. Jermiin et al. [32] also confirmed that when using phylogenetic methods based on site-homogenous models, it was difficult to recover correct tree topologies when datasets present compositional heterogeneity. However, at the same time, increasing the number of ingroup taxa in the phylogenetic analysis will make it harder to evaluate the correct phylogenetic tree. However, in order to better clarify the phylogenetic relationship within planthoppers, increasing the taxa number and using large datasets in the future is inevitable. Hence, sequence compositional heterogeneity should be seen as a serious problem in inferring planthopper phylogenetics in the future. In our analysis, all datasets under the site-heterogeneous mixture model recovered the eight planthoppers families as monophyletic and generated nearly consistent tree topologies. Our analysis showed that the adequacy of model fit is a significant factor for mitogenomes phylogenomic analysis of planthoppers. Since Urban and Cryan [10] and Song and Liang [9], there have been no comprehensive phylogenetic analyses of planthoppers. The relationships among Fulgoroidea remain uncertain, and the phylogenetic relationships within planthoppers remain to be clarified. Further taxonomic revision and methodological exploration will probably be necessary to recover a stable and highly supported phylogenetic hypothesis of planthoppers.

## 3. Materials and Methods

### 3.1. Sample Collection

*Cerynia lineola* was collected in the Yunnan Province, China. *Cromna sinensis* and *Zecheuna tonkinensis* were collected in Hainan Province, China. All specimens were preserved in 100% ethanol at −20 °C to allow DNA extraction. Samples were identified based on external morphological and male genital characters according to Bourgoin [33], as observed with an Olympus SZX10 stereomicroscope. All specimens and vouchers were placed in the Entomological Museum, Northwest A&F University, Yangling, Shaanxi, China.

### 3.2. DNA Extraction, Mitogenome Sequencing, Analysis, and Annotation

Genomic DNA was extracted from thorax and leg tissues using a TIANamp Genomic DNA Kit (TIANGEN, Beijing, China) following the manufacturer’s protocol. The whole genomic DNA was sequenced using the Illumina Miseq platform. The quality of data was checked by FastQC (Cambridge, UK) [34]. The adapters of raw data were excluded by AdapterRemoval version 2 (Copenhagen, Denmark) [35]. The de novo assembly of the mitochondrial genomes was performed using A5-miseq version 2.0 (New South Wales, Australia) [36]. The protein-coding genes (PCGs) were identified by the open reading frame (ORF) finder based on the invertebrate mitochondrial genetic code and alignment with the mitogenomes of *Geisha distinctissima* in Geneious v 11.0.2 (Auckland, New Zealand). The location and secondary structure of tRNAs were predicted using the MITOS Web Server (Leipzig, Germany) [37] with a 05-invertebrate mitochondrial gene code. The rRNA genes (*rrnS* and *rrnL*) and control region (A + T-rich region) were identified by the boundary of the adjacent tRNA genes (*trnL1* and *trnV*) and aligned with the mitogenomes of *Geisha distinctissima*. The mitogenome maps were produced using GCview (Alberta, Canada) [38].

Base composition, composition skew, codon usage, relative synonymous codon usage (RSCU), and architecture tables were analyzed using PhyloSuite v 1.2.2 (Wuhan, China) [39]. The tandem repeats of the control region (A + T-rich region) were identified by the Tandem Repeats Finder Online Server [40]. The nonsynonymous substitution rate (Ka) and synonymous substitution rate (Ks) of aligned protein-coding genes (PCGs) were calculated by DnaSP v 5.0 (Barcelona, Spain) [41].

### 3.3. Mitogenome Sequence Alignment and Analyses of Sequence Heterogeneity

Three newly sequenced Flatidae mitogenomes and 31 planthopper species from the NCBI database were selected as ingroups, representing eight families (13 species of Delphacidae, four species of Fulgoridae, one species of Derbidae, five species of Achilidae, one species of Lophopidae, three species of Issidae, three species of Ricaniidae, and four species of Flatidae). *Cicadetta abscondita* (Cicadidae), *Macrosteles quadrilineatus* (Cicadellidae), and *Philaenus spumarius* (Aphrophoridae) were selected as outgroups (Table 1). PhyloSuite v 1.2.2 [39] was used to extract 13 PCGs and two rRNAs. Each PCG was aligned based on codons for amino acids in MAFFT 7 (Osaka, Japan) [42]. Two rRNAs were aligned with individuals in the MAFFT 7 online service using the Q-INS-I algorithm (Osaka, Japan) [42]. All ambiguously aligned sites from 13 PCGs and two rRNAs were removed by GBlocks v.0.91b (Kyoto, Japan) [43]. For quality, all alignments were then checked and corrected manually in MEGA 7 (Tokyo, Japan) [44].

Five datasets were assembled for phylogenetic analysis: (1) the PCG matrix, including all three codon positions of PCGs (=10,776 bp); (2) the PCG12 matrix, only including the first and the second codon positions of PCGs (=7184 bp); (3) the PCGRNA matrix, including all three codon position of PCGs and two rRNA genes (=12,221 bp); (4) the PCG12RNA matrix, only including the first and the second codon positions of PCGs and two rRNA genes (=8629 bp); (5) and the AA matrix, including amino acid sequences of PCGs (= 3363 amino acids). The heterogeneity of sequence divergence within five datasets was analyzed using AliGROOVE (Bonn, Germany) [45] with the default sliding window size. Indels in the nucleotide datasets were treated as ambiguous. The amino acid matrix was aligned using the BLOSUM62 substitution matrix. This metric established the pairwise sequence distance between individual terminals or subclades with terminals outside of the focal group. The obtained scoring distance between sequences was then compared with similarity over the entire data matrix. Values varied from −1 (distances are very different from the average for the entire data matrix) to +1 (distances match the average for the entire matrix). This provided an indirect measure of the heterogeneity of a given sequence in comparison with the full dataset.

### 3.4. Phylogenetic Analyses Based on Site-homogeneous and Site-heterogeneous Models

Phylogenetic analyses of five datasets using site-homogeneous models were reconstructed by the MrBayes 3.2.6 (Stockholm, Sweden) [46] for Bayesian inference (BI) and the maximum likelihood (ML) using IQ-TREE 1.6.5 (Canberra, Australia) [47]. The best partitioning schemes and evolution models of both BI and ML analyses were implemented using PartitionFinder 2.1.1 (Sydney, Australia) [48] with the greedy search algorithm and Bayesian information criterion (BIC). MrBayes 3.2.6 [46] was run on CIPRES Science Gateway (San Diego, CA, USA) [49] with four chains included in two runs of 5,000,000–15,000,000 Markov chain Monte Carlo (MCMC) generations, sampled every 1000 generations with a burn-in of the initial 25%. The average standard deviation of split frequencies was <0.01, suggesting that runs reach convergence. IQ-TREE implemented the following test to determine node support for the ML analysis: 1000 replicates for the ultrafast bootstrap (UFB) algorithm [50].

Phylogenetic reconstruction of all datasets based on site-heterogeneous model CAT + GTR was performed using PhyloBayes MPI v1.5a on CIPRES [49]. Two independent trees searched and terminated when the likelihood of the sampled trees had stabilized, and the two runs reached convergence (maxdiff < 0.3 and minimum effective size > 50). The initial 25% of each run was discarded as burn-in, and a consensus tree was then generated from the remaining trees combined from two runs.

## 4. Conclusions

In this study, three flatid mitogenomes were newly sequenced, among which were representatives from the tribe Ceryniini (*Cerynia lineola*), Lawaini (*Cromna sinensis*), and Selizini (*Zecheuna tonkinensis*). Results showed the sequenced gene arrangements were consistent with the ancestral insect mitogenomes as understood today. Comparative analysis can improve our understanding of the evolution of flatid mitogenomes.

The results corroborated the monophyly of the eight families within Fulgoroidea and also showed that mitogenome sequences are effective molecular markers to study the phylogenetic relationships within Fulgoroidea. We consistently recovered Delphacidae as the sister group to the remaining families and Lophopidae with (Issidae + (Ricaniidae + Flatidae) forming a clade in all analyses. This site-heterogeneous model analysis was applied to planthopper phylogenetic analysis for the first time based on mitogenome data. Our results indicated that the mitogenomes of Delphacidae and Derbidae present more heterogeneity and a higher evolutionary rate and A + T content than found in other planthopper groups, which may be the cause of Delphacidae and Derbidae tending to fall together at the basal position on trees under site-homogenous models. Increased taxon sampling plus nuclear genes in planthoppers may further verify the influence of sequence compositional heterogeneity and clarify optimal strategies to pursue planthopper mitochondrial phylogenomics.

## Figures and Tables

**Table 1:** Taxa used in this study.

Superfamily	Family	Species	GenBank Number	References
Outgroups				
Membracoidea	Cicadellidae	*Macrosteles quadrilineatus*	NC_034781	[51]
Cercopoidea	Aphrophoridae	*Philaenus spumarius*	NC_005944	[52]
Cicadoidea	Cicadidae	*Cicadetta abscondita*	MW123088	Unpublished
Ingroup				
Fulgoroidea	Delphacidae	*Ugyops* sp.	MH352481	[53]
		*Sogatella vibix*	NC_042180	Unpublished
		*Peregrinus maidis*	NC_037182	[54]
		*Changeondelphax velitchkovskyi*	NC_037181	[55]
		*Nilaparvata bakeri*	NC_033388	Unpublished
		*Nilaparvata muiri*	NC_024627	[56]
		*Sogatella furcifera*	NC_021417	[57]
		*Laodelphax striatellus*	MK292897	[58]
		*Nilaparvata* sp.	KY039125	[59]
		*Nilaparvata lugens*	JN563995	[56]
		*Neomegamelanus elongatus*	MK251068	[20]
		*Saccharosydne procerus*	NC_042179	Unpublished
		*Saccharosydne saccharivora*	MK251072	[20]
	Fulgoridae	*Pyrops candelaria*	FJ006724	[60]
		*Aphaena amabilis*	NC_045075	[13]
		*Aphaena discolor*	MN025523	[13]
		*Lycorma delicatula*	NC_012835	[61]
	Achilidae	*Paracatonidia* sp.	MH324931	[14]
		*Betatropis formosana*	MH324927	[14]
		*Magadhaideus* sp.	MH324928	[14]
		*Plectoderini* sp.	MH324930	[14]
		*Peltatavertexalis horizontalis*	MH324929	[14]
	Derbidae	*Lydda* sp.	KY039126	[59]
	Lophopidae	*Lophops carinata*	MT990448	Unpublished
	Issidae	*Hemisphaerius rufovarius*	MT210096	[15]
		*Sivaloka damnosus*	FJ360694	[27]
		*Sivaloka* sp.	KY039137	[59]
	Ricaniidae	*Ricania shantungensis*	MT898421	Unpublished
		*Ricania marginalis*	NC_019597	[60]
		*Ricania speculum*	NC_031369	[62]
	Flatidae	*Cerynia lineola*	MW872011	Present study
		*Cromna sinensis*	MW872012	Present study
		*Geisha distinctissima*	NC_012617	[12]
		*Zecheuna tonkinensis*	MW872013	Present study

## Data Availability

Data is contained within the article or Supplementary Material.

## Supplementary Materials

The following are available online at https://www.mdpi.com/article/10.3390/ijms22115586/s1.
