# Supplementary material for: Characterization of Three Complete Mitogenomes of Flatidae (Hemiptera: Fulgoroidea) and Compositional Heterogeneity Analysis in the Planthoppers’ Mitochondrial Phylogenomics
Source: Int J Mol Sci. 2021 May 25;22(11):5586. doi: 10.3390/ijms22115586 (PMC8197536; doi:10.3390/ijms22115586)
Supplement: Supplementary file 1 [file ijms-22-05586-s001.zip › ijms-1203872-supplementary.pdf]

Characterization of Three Complete Mitogenomes of Flatidae (Hemiptera: Fulgoroidea) and compositional Heterogeneity Analysis in the Planthoppers' Mitochondrial Phylogenomics

Deqiang Ai[1], Lingfei Peng[2], Daozheng Qin[1,*], Yalin Zhang[1,*]

[1] Key Laboratory of Plant Protection Resources & Pest Management of the Ministry of Education, College of Plant Protection, Northwest A&F University, Yangling, Shaanxi 712100, China; aideqiang@nwafu.edu.cn (D.A.)
[2] Key Laboratory of Integrated Pest Management for Fujian-Taiwan Crops, Ministry of Agriculture, Fujian Agriculture and Forestry University, Fuzhou, Fujian 350002, China; lingfeipeng@fafu.edu.cn (L.P.)
* Correspondence: yalinzh@nwsuaf.edu.cn (Y.Z.); qindaozh0426@aliyun.com (D.Q.)

Supplementary caption:

Table S1 Mitogenomic organization of *Cerynia lineola*.

Table S2 Mitogenomic organization of *Cromna sinensis*.

Table S3 Mitogenomic organization of *Zecheuna tonkinensis*.

Table S4 Nucleotide composition of mitogenomes of *Cerynia lineola*, *Cromna sinensis*, and *Zecheuna tonkinensis*.

Table S5 Comparison of Ka/Ks and A + T content.

Table S6 Best partitioning schemes and models based on different datasets for IQ-TREE analysis.

Table S7 Best partitioning schemes and models based on different datasets for MrBayes analysis.

Figure S1. Predicted secondary cloverleaf structure for the tRNAs of *Cerynia lineola*.

Figure S2. Predicted secondary cloverleaf structure for the tRNAs of *Cromna sinensis*.

Figure S3. Predicted secondary cloverleaf structure for the tRNAs of *Zecheuna tonkinensis*.

Figure S4. Heterogeneous sequence divergence within datasets PCGRNA, PCG12 and PCG12RNA of planthoppers mitogenomes.

Table S1

Mitogenomic organization of *Cerynia lineola*.

Gene	Position		Size(bp)	Intergenic nucleotides	Codon		Strand
	From	To			Start	Stop	
trnI	1	62	62				J
trnQ	62	130	69	-1			N
trnM	130	194	65	-1			J
nad2	195	1,160	966		ATA	TAA	J
trnW	1,159	1,223	65	-2			J
trnC	1,216	1,279	64	-8			N
trnY	1,283	1,347	65	3			N
cox1	1,349	2,884	1,536	1	ATG	TAA	J
trnL2	2,885	2,948	64				J
cox2	2,949	3,626	678		ATT	TAA	J
trnK	3,628	3,696	69	1			J
trnD	3,697	3,757	61				J
atp8	3,758	3,910	153		ATT	TAA	J
atp6	3,904	4,555	652	-7	ATG	T	J
cox3	4,556	5,338	783		ATG	TAA	J
trnG	5,338	5,401	64	-1			J
nad3	5,402	5,749	348		ATT	TAA	J
trnA	5,749	5,813	65	-1			J
trnR	5,815	5,874	60	1			J
trnN	5,875	5,939	65				J
trnS1	5,939	5,996	58	-1			J
trnE	6,000	6,064	65	3			J
trnF	6,063	6,125	63	-2			N
nad5	6,125	7,840	1,716	-1	ATT	TAA	N
trnH	7,857	7,919	63	16			N
nad4	7,929	9,242	1,314	9	ATT	TAG	N
nad4L	9,254	9,526	273	11	ATG	TAA	N
trnT	9,529	9,588	60	2			J
trnP	9,591	9,653	63	2			N
nad6	9,655	10,155	501	1	ATA	TAA	J
cytb	10,148	11,269	1,122	-8	ATG	TAA	J
trnS2	11,271	11,332	62	1			J
nad1	11,331	12,275	945	-2	ATG	TAA	N
trnL1	12,277	12,338	62	1			N
rrnL	12,339	13,550	1,212				N
trnV	13,551	13,622	72				N
rrnS	13,623	14,347	725				N
CR	14,348	16,053	1,706				J

Table S2

Mitogenomic organization of *Cromna sinensis*.

Gene	Position		Size(bp)	Intergenic nucleotides	Codon		Strand
	From	To			Start	Stop	
trnI	1	62	62				J
trnQ	62	130	69	-1			N
trnM	130	195	66	-1			J
nad2	196	1,161	966		ATT	TAA	J
trnW	1,160	1,222	63	-2			J
trnC	1,215	1,275	61	-8			N
trnY	1,279	1,341	63	3			N
cox1	1,344	2,879	1,536	2	ATG	TAA	J
trnL2	2,880	2,941	62				J
cox2	2,942	3,616	675		ATA	TAA	J
trnK	3,617	3,685	69				J
trnD	3,686	3,751	66				J
atp8	3,752	3,904	153		ATA	TAA	J
atp6	3,898	4,549	652	-7	ATG	T	J
cox3	4,550	5,332	783		ATG	TAA	J
trnG	5,332	5,393	62	-1			J
nad3	5,388	5,741	354	-6	ATG	TAA	J
trnA	5,751	5,814	64	9			J
trnR	5,817	5,876	60	2			J
trnN	5,882	5,943	62	5			J
trnS1	5,943	6,001	59	-1			J
trnE	6,003	6,067	65	1			J
trnF	6,066	6,127	62	-2			N
nad5	6,128	7,853	1,726		ATA	T	N
trnH	7,847	7,910	64	-7			N
nad4	7,913	9,235	1,323	2	ATA	TAA	N
nad4L	9,255	9,527	273	19	ATG	TAG	N
trnT	9,530	9,591	62	2			J
trnP	9,594	9,655	62	2			N
nad6	9,659	10,153	495	3	ATG	TAA	J
cytb	10,146	11,267	1,122	-8	ATG	TAG	J
trnS2	11,268	11,330	63				J
nad1	11,349	12,290	942	18	ATG	TAG	N
trnL1	12,292	12,353	62	1			N
rrnL	12,354	13,551	1,198				N
trnV	13,552	13,622	71				N
rrnS	13,623	14,362	740				N
CR	14,363	15,329	967				J

Table S3

Mitogenomic organization of *Zecheuna tonkinensis*.

Gene	Position		Size(bp)	Intergenic nucleotides	Codon		Strand
	From	To			Start	Stop	
trnI	1	67	67				J
trnQ	99	167	69	31			N
trnM	167	232	66	-1			J
nad2	233	1,198	966		ATT	TAA	J
trnW	1,208	1,272	65	9			J
trnC	1,283	1,343	61	10			N
trnY	1,355	1,417	63	11			N
cox1	1,425	2,963	1,539	7	ATG	TAA	J
trnL2	2,970	3,032	63	6			J
cox2	3,033	3,704	672		ATA	TAA	J
trnK	3,710	3,778	69	5			J
trnD	3,783	3,848	66	4			J
atp8	3,849	3,992	144		ATT	TAG	J
atp6	3,986	4,637	652	-7	ATG	T	J
cox3	4,638	5,420	783		ATG	TAA	J
trnG	5,437	5,499	63	16			J
nad3	5,500	5,847	348		ATA	TAA	J
trnA	5,849	5,913	65	1			J
trnR	5,919	5,981	63	5			J
trnN	6,006	6,070	65	24			J
trnS1	6,070	6,127	58	-1			J
trnE	6,137	6,198	62	9			J
trnF	6,211	6,279	69	12			N
nad5	6,316	7,938	1,623	36	ATG	TAA	N
trnH	7,940	8,003	64	1			N
nad4	8,023	9,270	1,248	19	ATG	TAG	N
nad4L	9,264	9,539	276	-7	ATG	TAA	N
trnT	9,542	9,608	67	2			J
trnP	9,619	9,684	66	10			N
nad6	9,686	10,180	495	1	ATA	TAA	J
cytb	10,185	11,306	1,122	4	ATG	TAA	J
trnS2	11,325	11,390	66	18			J
nad1	11,444	12,379	936	53	ATA	TAG	N
trnL1	12,380	12,445	66				N
rrnL	12,446	13,674	1,229				N
trnV	13,675	13,742	68				N
rrnS	13,743	14,474	732				N
CR	14,475	15,613	1,139				J

Table S4

Nucleotide composition of mitogenomes of *Cerynia lineola, Cromna sinensis,* and *Zecheuna tonkinensis*.

Regions	Size (bp)	T(U)%	C%	A%	G%	AT%	GC%	AT skew	GC skew
Cerynia lineola									
Full genomes	16,053	28.6	15.4	47.6	8.4	76.2	23.8	0.249	-0.292
PCGs	10,986	42.5	13.3	32.8	11.4	75.3	24.7	-0.129	-0.076
1st condon position	3,662	37.0	11.3	36.1	15.5	73.1	26.8	-0.013	0.157
2nd condon position	3,662	47.8	19.1	19.6	13.4	67.4	32.5	-0.418	-0.177
3rd condon position	3,662	42.7	9.3	42.7	5.3	85.4	14.6	-0.001	-0.277
tRNAs	1,406	35.3	10.0	40.3	14.5	75.6	24.5	0.066	0.186
rRNAs	1,937	50.3	7.2	28.7	13.8	79.0	21.0	-0.273	0.317
Control Region	1,706	34.2	13.9	45.1	6.8	79.3	20.7	0.137	-0.343
Cromna sinensis									
Full genomes	15,329	27.5	15.8	47.4	9.3	74.9	25.1	0.265	-0.258
PCGs	10,998	41.8	14.1	32.1	12.0	73.9	26.1	-0.131	-0.082
1st condon position	3,666	36.3	12.0	35.1	16.6	71.4	28.6	-0.018	-0.159
2nd condon position	3,666	48.1	19.6	19.6	12.6	67.7	32.2	-0.421	-0.218
3rd condon position	3,666	40.9	10.7	41.7	6.8	82.6	17.5	0.010	-0.225
tRNAs	1,399	35.5	10.7	40.0	13.9	75.5	24.6	0.060	0.128
rRNAs	1,938	49.1	7.9	28.1	14.9	77.2	22.8	-0.272	0.305
Control Region	967	33.4	13.0	47.2	6.4	80.6	19.4	0.171	-0.340
Zecheuna tonkinensis									
Full genomes	15,613	30.5	13.0	49.0	7.5	79.5	20.5	0.233	-0.267
PCGs	10,803	43.2	11.3	35.1	10.4	78.3	21.7	-0.104	-0.042
1st condon position	3,601	36.6	9.9	38.6	14.8	75.2	24.7	0.027	0.197
2nd condon position	3,601	48.1	18.8	20.3	12.7	68.4	31.5	-0.406	-0.192
3rd condon position	3,601	45.0	5.1	46.4	3.5	91.4	8.6	0.015	-0.184
tRNAs	1,431	37.1	8.6	43.0	11.3	80.1	19.9	0.073	0.137
rRNAs	1,961	49.8	7.2	30.3	12.6	80.1	19.8	-0.243	0.270
Control Region	1,139	36.0	12.1	47.6	4.3	83.6	16.4	0.139	-0.476

Table S5

Comparison of Ka/Ks and A + T content.

Family	Species	Ka/Ks[a]	A + T content[b]
Delphacidae	*Ugyops* sp.	0.611447	76.3
	Sogatella vibix	0.644155	75.3
	Peregrinus maidis	0.646251	75.6
	Changeondelphax velitchkovskyi	0.630288	74.3
	Nilaparvata bakeri	0.67709	76.5
	Nilaparvata muiri	0.645893	75.3
	Nilaparvata sp.	0.641439	75.4
	Nilaparvata lugens	0.667751	75.9
	Sogatella furcifera	0.621768	74.3
	Laodelphax striatellus	0.65374	75.6
	Neomegamelanus elongatus	0.643673	76.1
	Saccharosydne procerus	0.711141	79
	Saccharosydne saccharivora	0.68135	78.8
Fulgoridae	*Pyrops candelaria*	0.598085	72.6
	Aphaena amabilis	0.670371	77
	Aphaena discolor	0.640664	75.9
	Lycorma delicatula	0.62307	75.2
Achilidae	*Paracatonidia* sp.	0.578901	75.1
	Betatropis formosana	0.611749	76.1
	Magadhaideus sp.	0.571576	72.3
	Plectoderini sp.	0.583587	74
	Peltatavertexalis horizontalis	0.587692	74
Derbidae	*Lydda* sp.	0.670112	77.4
Lophopidae	*Lophops carinata*	0.639152	76.1
Issidae	*Hemisphaerius rufovarius*	0.619477	76.9
	Sivaloka damnosus	0.642221	75.6
	Sivaloka sp.	0.569616	75.3
Ricaniidae	*Ricania shantungensis*	0.573077	75
	Ricania marginalis	0.655543	76.1
	Ricania speculum	0.599573	73.4
Flatidae	*Cerynia lineola*	0.575224	75
	Cromna sinensis	0.589202	73.7
	Geisha distinctissima	0.593165	73.3
	Zecheuna tonkinensis	0.674622	78.2

[a]the Ka/Ks was calculated from the dataset PCG, using *Philaenus spumarius* as a reference; [b]the A + T content was calculated from the dataset PCG.

Table S6

Best partitioning schemes and models based on different datasets for IQ-TREE analysis.

Dataset	Partitioning scheme	Models
PCGRNA	P1: *nad3*_codon1, *cytb*_codon1, *atp6*_codon1, *cox3*_codon1, *cox2*_codon1	GTR+I+G
	P2: *cox1*_codon2, *cytb*_codon2, *cox2*_codon2, *atp6*_codon2, *cox3*_codon2	GTR+I+G
	P3: *atp6*_codon3, *cox2*_codon3, *cox3*_codon3	TIM+I+G
	P4: *nad2*_codon1, *nad6*_codon1, *atp8*_codon1	TRN+I+G
	P5: *atp8*_codon2, *nad6*_codon2, *nad3*_codon2, *nad2*_codon2	TVM+G
	P6: *nad3*_codon3, *nad6*_codon3, *atp8*_codon3	TRN+G
	P7: *cox1*_codon1	GTR+G
	P8: *cytb*_codon3, *cox1*_codon3	TVM+I+G
	P9: *nad1*_codon1, *nad4L*_codon1, *nad5*_codon1, *nad4*_codon1	GTR+I+G
	P10: *nad4L*_codon2, *nad1*_codon2, *nad4*_codon2, *nad5*_codon2	GTR+I+G
	P11: *nad1*_codon3, *nad4*_codon3, *nad4L*_codon3, *nad5*_codon3	TVM+G
	P12: *nad2*_codon3	K81UF+G
	P13: *rrnL*, *rrnS*	GTR+I+G
PCG	P1: *nad3*_codon1, *cytb*_codon1, *atp6*_codon1, *cox3*_codon1, *cox2*_codon1	GTR+I+G
	P2: *cox1*_codon2, *cox2*_codon2, *cytb*_codon2, *atp6*_codon2, *cox3*_codon2	GTR+I+G
	P3: *atp6*_codon3, *cox2*_codon3, *cox3*_codon3	TIM+I+G
	P4: *nad2*_codon1, *nad6*_codon1, *atp8*_codon1	TRN+I+G
	P5: *atp8*_codon2, *nad6*_codon2, *nad3*_codon2, *nad2*_codon2	TVM+G
	P6: *nad3*_codon3, *atp8*_codon3, *nad6*_codon3	TRN+G
	P7: *cox1*_codon1	GTR+G
	P8: *cytb*_codon3, *cox1*_codon3	TVM+I+G
	P9: *nad1*_codon1, *nad4L*_codon1, *nad5*_codon1, *nad4*_codon1	GTR+I+G
	P10: *nad4L*_codon2, *nad1*_codon2, *nad4*_codon2, *nad5*_codon2	GTR+I+G
	P11: *nad1*_codon3, *nad4*_codon3, *nad4L*_codon3, *nad5*_codon3	TVM+G
	P12: *nad2*_codon3	K81UF+G
PCG12RNA	P1: *cytb*, *cox2*, *atp6*, *cox3*	GTR+I+G
	P2: *nad6*, *nad3*, *atp8*, *nad2*	GTR+I+G
	P3: *cox1*	GTR+I+G
	P4: *nad1*, *nad4L*, *nad5*, *nad4*	GTR+I+G
	P5: *rrnS*, *rrnL*	GTR+I+G
PCG12	P1: *cytb*, *cox2*, *atp6*, *cox3*	GTR+I+G
	P2: *nad6*, *nad3*, *atp8*, *nad2*	GTR+I+G
	P3: *cox1*	GTR+I+G
	P4: *nad1*, *nad4L*, *nad4*, *nad5*	GTR+I+G
AA	P1: *cytb*, *cox2*, *cox3*, *atp6*, *nad2*, *atp8*, *nad6*, *nad3*	MTART+I+G+F
	P2: *cox1*	MTART+I+G
	P3: *nad4L*, *nad4*, *nad1*, *nad5*	MTART+I+G+F

Table S7

Best partitioning schemes and models based on different datasets for MrBayes analysis.

Dataset	Partitioning scheme	Models
PCGRNA	P1: *atp8*_codon1, *atp6*_codon1, *nad3*_codon1	GTR+I+G
	P2: *cox1*_codon2, *cox2*_codon2, *cytb*_codon2, *cox3*_codon2, *atp6*_codon2	GTR+I+G
	P3: *cytb*_codon3, *cox1*_codon3, *cox2*_codon3, *cox3*_codon3, *atp6*_codon3	GTR+I+G
	P4: *atp8*_codon2, *nad2*_codon2, *nad6*_codon2, *nad3*_codon2	GTR+G
	P5: *nad3*_codon3, *atp8*_codon3, *nad6*_codon3	HKY+G
	P6: *cox1*_codon1	GTR+G
	P7: *cytb*_codon1, *cox3*_codon1, *cox2*_codon1	GTR+I+G
	P8: *nad1*_codon1, *nad4L*_codon1, *nad4*_codon1, *nad5*_codon1	GTR+I+G
	P9: *nad4L*_codon2, *nad1*_codon2, *nad5*_codon2, *nad4*_codon2	GTR+I+G
	P10: *nad1*_codon3, *nad4*_codon3, *nad5*_codon3, *nad4L*_codon3	GTR+G
	P11: *nad6*_codon1, *nad2*_codon1	HKY+I+G
	P12: *nad2*_codon3	HKY+G
	P13: *rrnL*, *rrnS*	GTR+I+G
PCG	P1: *nad3*_codon1, *atp6*_codon1, *cytb*_codon1, *cox3*_codon1, *cox2*_codon1	GTR+I+G
	P2: *cox1*_codon2, *cox2*_codon2, *cytb*_codon2, *cox3*_codon2, *atp6*_codon2	GTR+I+G
	P3: *cytb*_codon3, *cox1*_codon3, *cox2*_codon3, *cox3*_codon3, *atp6*_codon3	GTR+I+G
	P4: *atp8*_codon1, *nad2*_codon1, *nad6*_codon1	HKY+I+G
	P5: *atp8*_codon2, *nad2*_codon2, *nad6*_codon2, *nad3*_codon2	GTR+G
	P6: *nad3*_codon3, *atp8*_codon3, *nad6*_codon3	HKY+G
	P7: *cox1*_codon1	GTR+G
	P8: *nad1*_codon1, *nad4L*_codon1, *nad5*_codon1, *nad4*_codon1	GTR+I+G
	P9: *nad4L*_codon2, *nad1*_codon2, *nad4*_codon2, *nad5*_codon2	GTR+I+G
	P10: *nad1*_codon3, *nad4*_codon3, *nad5*_codon3, *nad4L*_codon3	GTR+G
	P11: *nad2*_codon3	HKY+G
PCG12RNA	P1: *atp6*, *cytb*, *cox3*, *cox2*	GTR+I+G
	P2: *atp8*, *nad2*, *nad6*, *nad3*	GTR+I+G
	P3: *cox1*	GTR+I+G
	P4: *nad1*, *nad4L*, *nad4*, *nad5*	GTR+I+G
	P5: *rrnS*, *rrnL*	GTR+I+G
PCG12	P1: *atp6*, *cytb*, *cox3*, *cox2*	GTR+I+G
	P2: *atp8*, *nad2*, *nad6*, *nad3*	GTR+I+G
	P3: *cox1*	GTR+I+G
	P4: *nad1*, *nad4L*, *nad4*, *nad5*	GTR+I+G
AA	P1: *atp6*, *cox3*, *cytb*, *cox2*	MTREV+I+G
	P2: *nad4L*, *nad2*, *atp8*	MTREV+I+G
	P3: *cox1*	MTREV+I+G
	P4: *nad1*, *nad6*, *nad3*	MTREV+G
	P5: *nad5*, *nad4*	MTREV+I+G

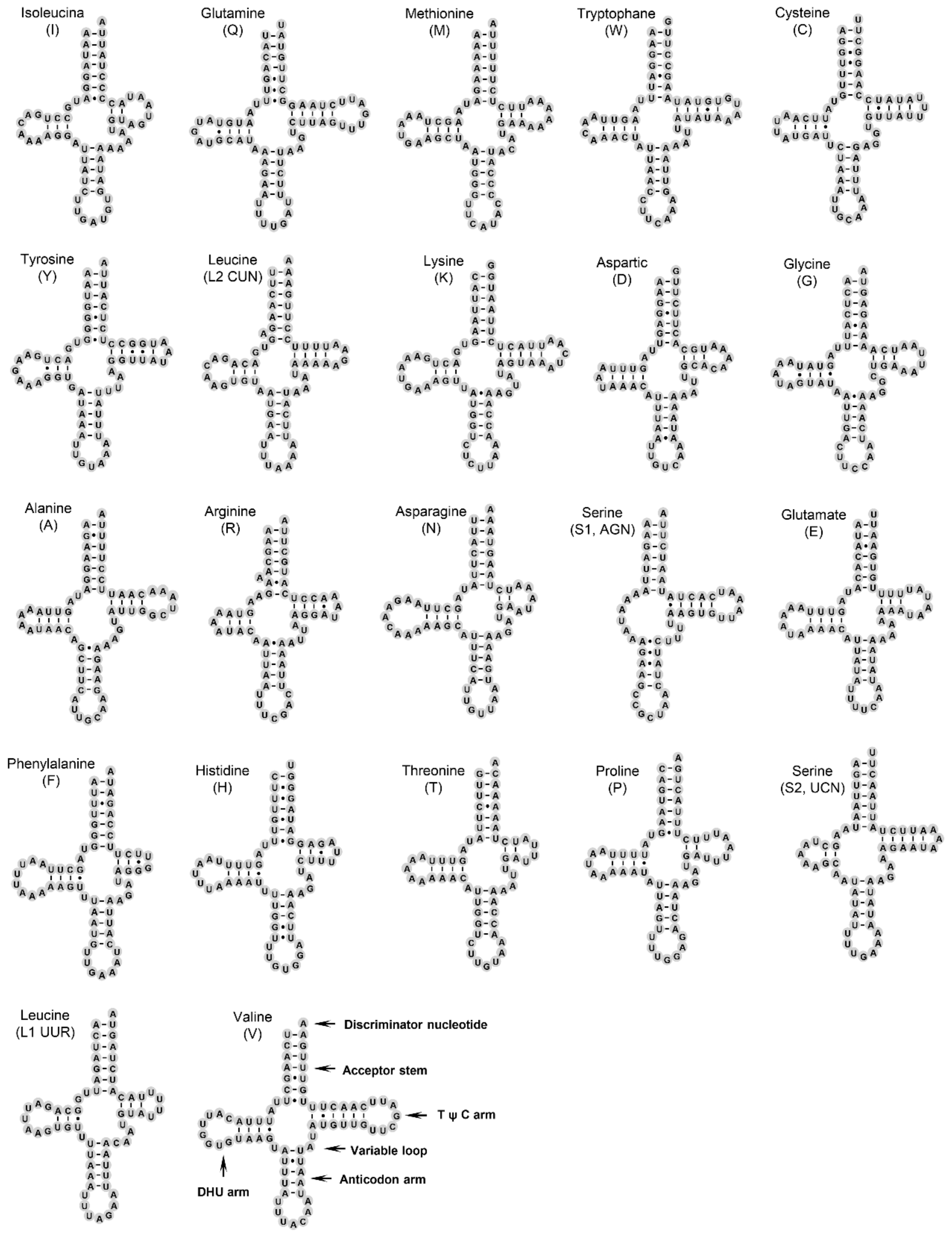

Figure S1. Predicted secondary cloverleaf structure for the tRNAs of *Cerynia lineola*

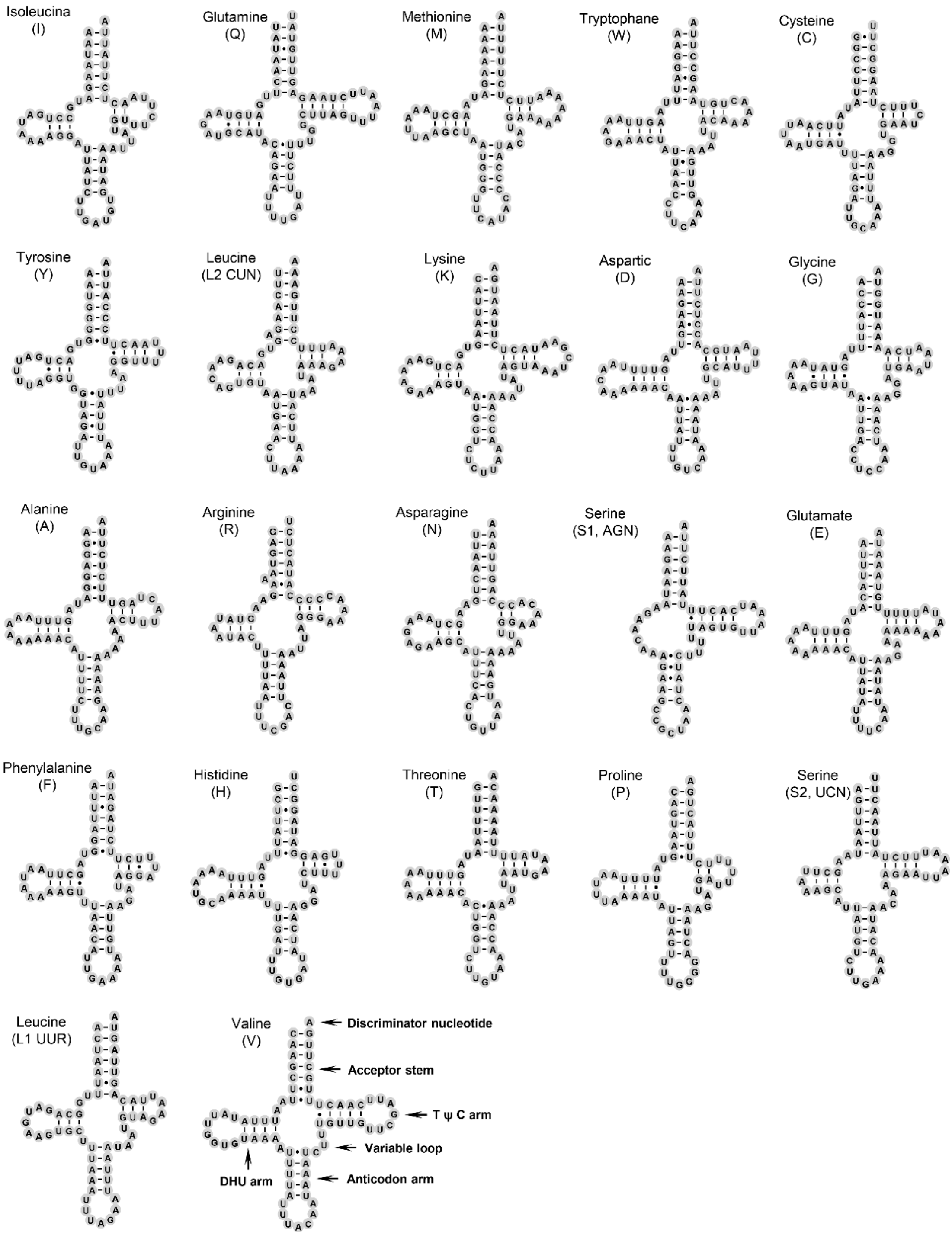

Figure S2. Predicted secondary cloverleaf structure for the tRNAs of *Cromna sinensis*

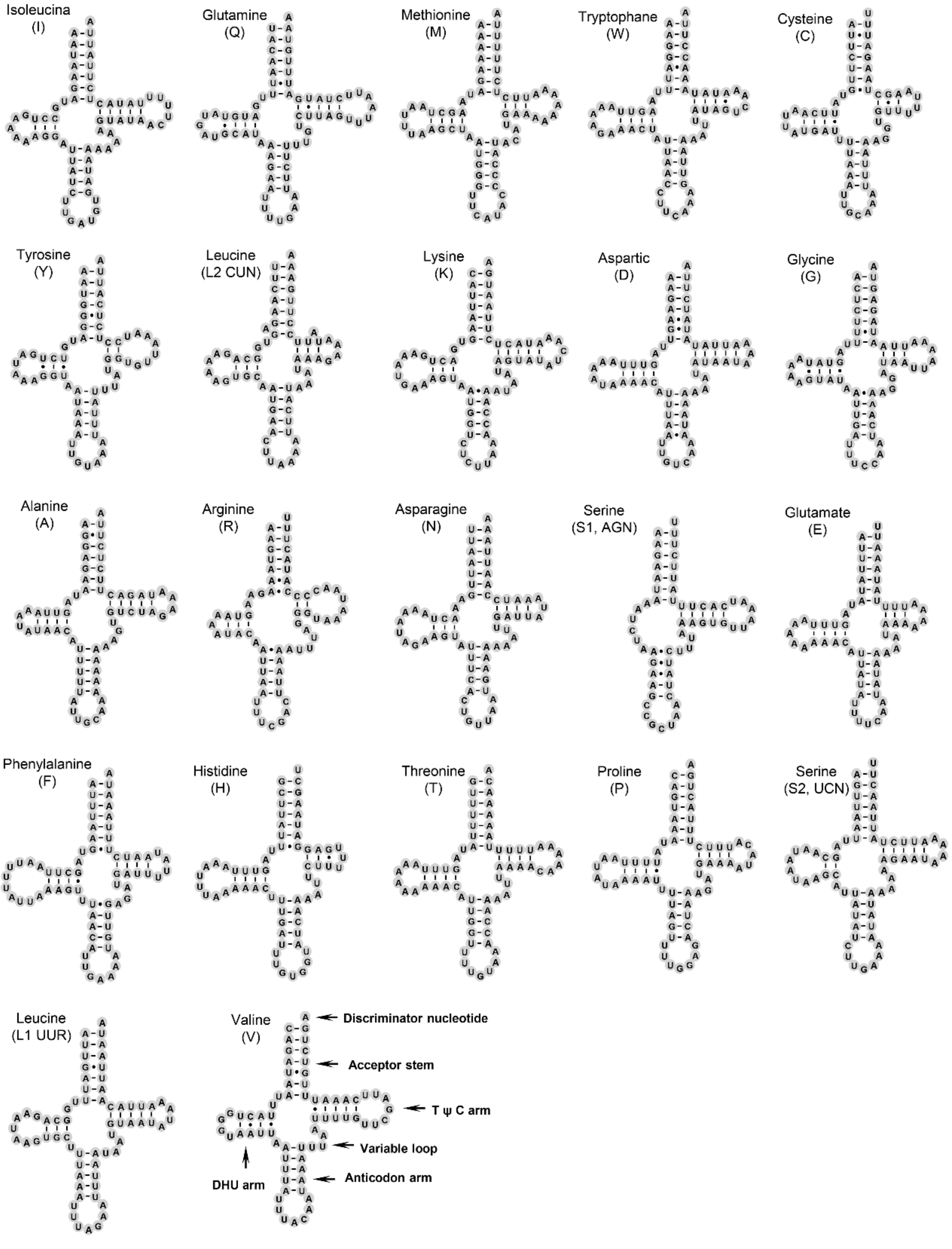

Figure S3. Predicted secondary cloverleaf structure for the tRNAs of *Zecheuna tonkinensis*

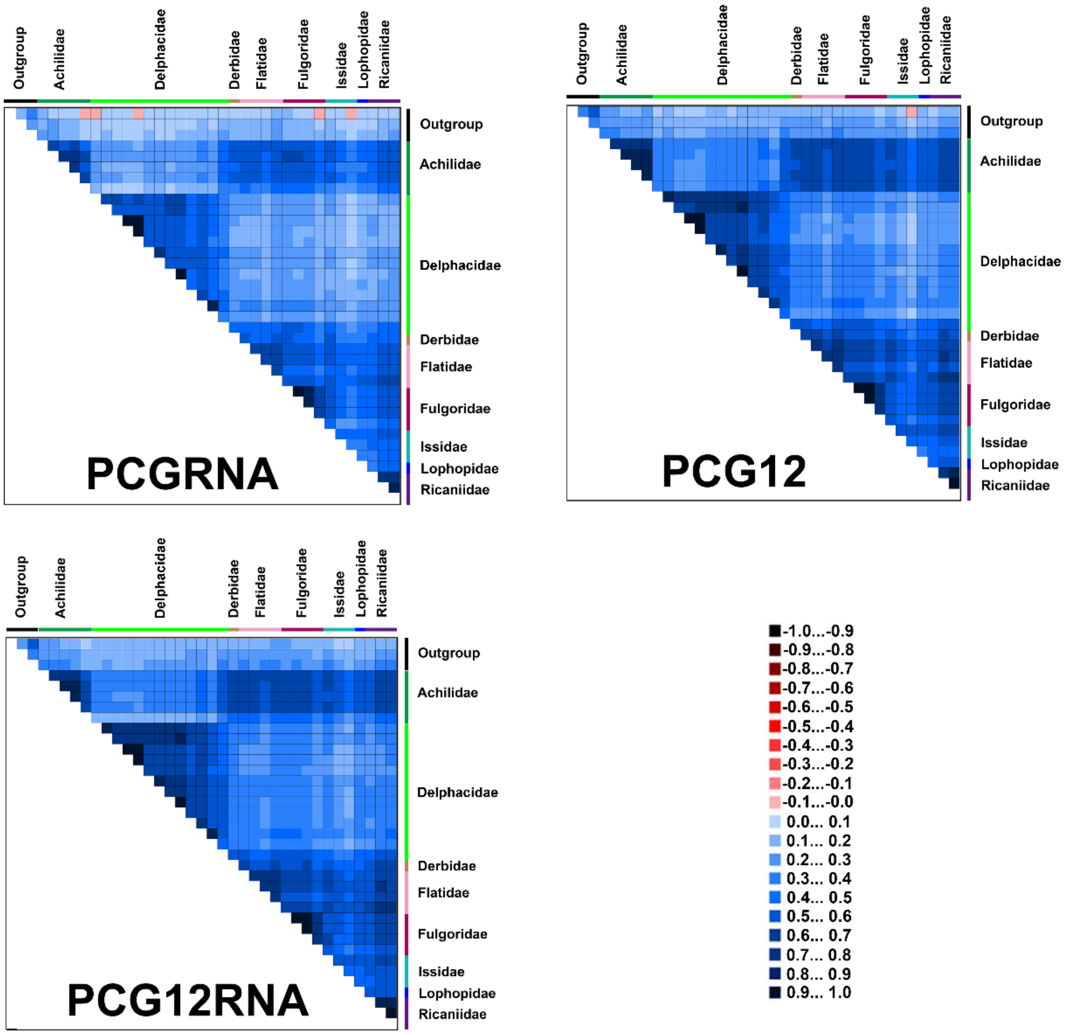

Figure S4. Heterogeneous sequence divergence within datasets PCGRNA, PCG12 and PCG12RNA of planthopper mitogenomes.
